# Cytomegalovirus DNAemia in Hospitalized Adults With SARS-CoV-2 Infection Requiring Supplemental Oxygen: Virologic and Clinical Characteristics and Association With Outcomes

**DOI:** 10.1093/infdis/jiaf649

**Published:** 2025-12-31

**Authors:** Michael Boeckh, Hu Xie, Terry Stevens-Ayers, Linda Sircy, Danniel Zamora, Jason D Goldman, Christopher W Woods, Renee D Stapleton, Gordon Rubenfeld, Andre Kalil, Keith R Jerome, Sayan Dasgupta, Ajit P Limaye

**Affiliations:** Vaccine and Infectious Disease Division, Fred Hutchinson Cancer Center, Seattle, Washington, USA; Department of Medicine, University of Washington, Seattle, Washington, USA; Vaccine and Infectious Disease Division, Fred Hutchinson Cancer Center, Seattle, Washington, USA; Vaccine and Infectious Disease Division, Fred Hutchinson Cancer Center, Seattle, Washington, USA; Vaccine and Infectious Disease Division, Fred Hutchinson Cancer Center, Seattle, Washington, USA; Department of Medicine, City of Hope Medical Center, Duarte, California, USA; Department of Medicine, University of Washington, Seattle, Washington, USA; Infectious Disease, Providence Swedish Medical Center, Seattle, Washington, USA; Division of Infectious Diseases, Center for Precision Health, Duke University, Durham, North Carolina, USA; Department of Medicine, University of Vermont, Burlington, Vermont, USA; Department of Medicine, University of Toronto, Toronto, Ontario, Canada; Division of Infectious Diseases, University of Nebraska, Lincoln, Nebraska, USA; Vaccine and Infectious Disease Division, Fred Hutchinson Cancer Center, Seattle, Washington, USA; Department of Laboratory Medicine and Pathology, University of Washington, Seattle, Washington, USA; Vaccine and Infectious Disease Division, Fred Hutchinson Cancer Center, Seattle, Washington, USA; Infectious Disease Division, University of California San Francisco, San Francisco, California, USA

**Keywords:** cytomegalovirus, SARS-CoV-2, viremia, viral shedding, recovery

## Abstract

**Background:**

Cytomegalovirus (CMV) reactivation occurs in the context of coronavirus disease 2019 (COVID-19); however, the viral kinetics, risk factors, and clinical outcomes are poorly defined.

**Methods:**

We examined the association of CMV DNAemia with clinical outcomes among participants of a randomized trial of remdesivir with or without baricitinib (National Institute of Allergy and Infectious Diseases [NIAID], Adaptive COVID-19 Treatment Trial 2 [ACTT-2]). Plasma CMV DNAemia from CMV-seropositive participants with COVID-19 (NIAID ordinal scale [OS] 5, 6, or 7 at entry) were assessed longitudinally by quantitative polymerase chain reaction. Factors associated with CMV DNAemia, and clinical outcomes were analyzed by Cox regression and proportional odds models.

**Results:**

Of 772 trial participants with available samples, 643 (83%) were CMV seropositive. Baseline CMV serostatus was not associated with COVID-19 outcomes. The cumulative incidence of CMV DNAemia among seropositive persons by day 28 was overall 11% (baseline OS 5, 6.3%; OS 6, 16.4%; OS 7, 24.7%), and was associated with older age, baseline OS, male sex, lymphopenia, and systemic corticosteroid use, while remdesivir and baricitinib did not affect risk. CMV DNAemia was associated with a lower probability of improvement by day 29 (adjusted hazard ratio, 0.3 [95% confidence interval, .17–.56]), with a more pronounced delay of recovery with higher CMV viral load. CMV DNAemia was also associated with higher severe acute respiratory syndrome coronavirus 2 (SARS-CoV-2) viral load and death.

**Conclusions:**

In hospitalized adults with COVID-19 requiring oxygen, CMV viremia occurs within well-defined clinical risks and is independently associated with delayed recovery from illness, higher SARS-CoV-2 viral load, and increased mortality.

## Teaser

Cytomegalovirus (CMV)–seropositive participants requiring supplemental oxygen were assessed for CMV DNAemia over 30 days. CMV reactivation occurred in 11% of participants and was associated with a delay in clinical recovery, increased SARS-CoV-2 viral load, and mortality.

**(See the Editorial Commentary by Haidar on pages 799–801.)**

Coronavirus disease 2019 (COVID-19) continues to cause hospitalization, critical illness, and death at rates similar to or in excess of influenza in 2025 [1, 2]. Most severe cases occur in individuals aged >60 years, unvaccinated persons, and those with underlying conditions [3]. An association of cytomegalovirus (CMV) reactivation with worse outcomes has been reported among adults with multiple etiologies of critical illness [4] but limited data are available for COVID-19 [5]. The epidemiologic pattern of COVID-19 morbidity and mortality is highly consistent with the hypothesis that CMV is a disease-modifying factor. CMV seroprevalence increases with age [6], accelerates immunosenescence [7], and increases death rates from vascular disease [8–10]. Mechanistically, the initial insult of severe acute respiratory syndrome coronavirus 2 (SARS-CoV-2) infection may lead to a relative state of immunosuppression characterized by lymphopenia and thrombocytopenia [11–13]. Dysregulated cytokine networks [14–16] may lead to reactivation of CMV from latency, which might further alter the inflammatory response and affect outcomes [17].

CMV has been shown to exert indirect immunomodulatory effects in transplant recipients by affecting macrophage and cytokine responses as well as T-cell immunity [18]. Also, high-grade CMV reactivation can cause lung tissue injury with direct damage to target organs (eg, pneumonia) in immunocompromised patients [19, 20]. There are increasing data supporting that CMV end organ disease can also occur in critically ill immunocompetent patients with and without COVID-19 [21–26]; however, a systematic longitudinal analysis of the virologic characteristics, risk factors, and associated clinical outcomes is lacking [27].

Here we examine the risk factors and kinetics of CMV DNAemia and their impact on clinical and virologic outcomes among patients hospitalized for SARS-CoV-2 pneumonia who participated in a large prospective randomized trial that provided standardized sampling and clinical outcomes [28].

## METHODS

### Study Design

The study examined stored samples and data from a randomized placebo-controlled trial of remdesivir plus baricitinib (Adaptive COVID-19 Treatment Trial 2, or placebo [ACTT-2], NCT04401579) [28]. Samples and data were made available by the National Institute of Allergy and Infectious Diseases (NIAID). For this analysis, we included SARS-CoV-2–infected patients (aged ≥18 years) who required supplemental oxygen (NIAID ordinal scale [OS] category 5), noninvasive ventilation or high-flow oxygen devices (OS category 6), or invasive mechanical ventilation or extracorporeal membrane oxygenation (OS category 7) (Supplementary Table 1) with available serum samples in the sample repository (772 of 891 randomized OS 5–7 participants [86.6%]).

### CMV Serostatus and DNAemia Testing

After receipt of samples from NIAID, we first established CMV serostatus by a standard immunoglobulin G (IgG) assay (LIAISON CMV IgG Assay, Diasorin, Stillwater, MN, USA). In CMV-seropositive trial participants, baseline and longitudinal plasma samples were tested by quantitative CMV plasma polymerase chain reaction (PCR; Abbott RealTime CMV assay, Abbott Laboratories, Chicago, IL, USA). The limit of detection is 31.2 IU/mL and the quantitation threshold is 50 IU/mL for this PCR assay [29, 30]. Samples were collected at baseline (study enrollment) and on days 3, 5, 8, 11, 15, and 29 (end of study).

### Definitions and Outcomes

All endpoints and baseline variables were obtained from the original data set [28] and used without modification. Lymphopenia was defined as <1000 cells/μL and further categorized by lower thresholds. CMV DNAemia was defined as any detectable viremia above the limit of detection; high-level viremia was defined as a ≥100 IU/mL. SARS-CoV-2 shedding was determined by PCR of oropharyngeal, nasopharyngeal, or nasal swabs. SARS-CoV-2 shedding rate was defined as number of days with detectable SARS-CoV-2 divided by the number of days of follow-up. Shedding was assumed for the days following a positive test until the next test or a 7-day carry-forward period, whichever was shorter. As per the parent study, time to clinical recovery was defined as the number of days to obtain NIAID OS 1–3 status, and improvement by day 15 or day 29 was defined as a decrease by at least 1 category or more on the NIAID OS.

### Statistical Analysis

Fine and Gray versions of multivariable Cox proportional hazard regression models were used to evaluate risk factors associated with CMV DNAemia and outcomes of clinical recovery, improvement, and mortality. CMV viremia was analyzed both as detection at any level and at thresholds of ≥100, >500, >1000 IU/mL as measurements of higher viral load, treating death as a competing risk. The impact of CMV viremia on mortality was assessed by multivariable Cox regression models among all patients at day 29 as well as day 15 survivors (randomization arm of the original trial was included in all analyses). The cumulative incidence curves were generated to estimate probabilities of CMV reactivation and Kaplan–Meier methods were used to estimate incidence of overall mortality. Clinical recovery status at day 15 and 29 was analyzed using proportional odds models. Landmark analyses were conducted to address immortal time bias. Covariates included in all statistical models are listed in Table 1.

**Table 1.** Patient Characteristics in the Original Cohort and Subsets Studied in This Analysis

Characteristic	Original Cohort (OS 5, 6, or 7) (n = 891)	Participants With Available Samples (n = 772)	CMV-Seropositive Participants^a^ (n = 643)
Age, y, median (range)	56.00 (18.00–89.00)	56.00 (18.00–89.00)	56.00 (18.00–89.00)
Randomized treatment			
Baricitinib + remdesivir	445 (50)	388 (50)	326 (51)
Placebo + remdesivir	446 (50)	384 (50)	317 (49)
BMI, kg/m^2^, median (range)	31.00 (16.10–91.90)	31.15 (16.10–91.90)	31.00 (16.10–91.90)
Sex			
Female	325 (36)	289 (37)	247 (38)
Male	566 (64)	483 (63)	396 (62)
Race			
White	438 (49)	371 (48)	286 (44)
Native American/Hawaiian	17 (2)	16 (2)	13 (2)
Asian	64 (7)	39 (5)	36 (6)
Black or African	133 (15)	121 (16)	102 (16)
Unknown	239 (27)	225 (29)	206 (32)
Ethnicity			
Hispanic or Latino	481 (54)	436 (56)	390 (61)
Not Hispanic	395 (44)	322 (42)	244 (38)
Not reported/unknown	15 (2)	14 (2)	9 (1)
Geographic region			
Non-US site	113 (13)	85 (11)	80 (12)
US site	778 (87)	687 (89)	563 (88)
Disease			
Baseline severity OS score			
5	564 (63)	492 (64)	399 (62)
6	216 (24)	186 (24)	159 (25)
7	111 (12)	94 (12)	85 (13)
Duration of symptoms at randomization, median d (range)	8.00 (0.00–35.00)	8.00 (0.00–35.00)	8.00 (0.00–35.00)
Corticosteroid use at baseline			
No	854 (96)	735 (95)	611 (95)
Yes	37 (4)	37 (5)	32 (5)
ALC at baseline, cells/μL			
0–500	83 (10)	72 (9)	60 (9)
>500–1000	363 (42)	323 (42)	271 (42)
>1000–1500	269 (31)	229 (30)	182 (28)
>1500	154 (18)	139 (18)	123 (19)
Missing	4 (<1)	4 (1)	3 (<1)

Data are presented as No. (%) unless otherwise indicated.

Abbreviations: ALC, absolute lymphocyte count; BMI, body mass index; CMV, cytomegalovirus; OS, ordinal scale; US, United States.

^a^Among participants with available samples.

To assess the impact of CMV serostatus on SARS-CoV-2, linear regression models were used to compare the CMV shedding rate. The impact of CMV DNAemia on SARS-CoV-2 shedding duration and peak viral loads was analyzed by linear regression models among day 15 survivors. Two-sided *P* values <.05 were considered statistically significant. All statistical analyses were performed using SAS 9.4 TS1M6 for Windows (SAS Institute, Cary, NC, USA).

## RESULTS

### Study Cohort

Characteristics of the original cohort (OS 5, 6, 7), the subset of participants with available samples, and those who were CMV seropositive are shown in Table 1. Of the 891 persons with OS 5–7 in the parent study, 772 (87%) had available samples. Overall, the participants included in this study were similar to the overall parent study. Most were male (63%), obese (median BMI, 31 kg/m^2^), and enrolled in the United States. The vast majority (95%) of persons were treated with dexamethasone at baseline. The impact of the OS groups on outcome for included participants was similar to the parent trial (Supplementary Figure 1). Characteristics for subset analyses are shown in Supplementary Table 2.

### Impact of CMV Serostatus on Outcomes

CMV seropositivity at baseline occurred in 643 of 772 participants (83%). CMV seropositivity at baseline was associated with delayed clinical recovery by day 29 in univariate analysis (hazard ratio [HR], 0.77 [95% confidence interval {CI}, .63–.93]), but the association was no longer statistically significant in models adjusted for age, OS group, lymphopenia at baseline, randomization group, ethnicity, and BMI (data not shown). CMV seropositivity did not affect time to recovery in the multivariate model: HR for time to recover was 0.91 (95% CI, .73–1.12; *P* = .37 (Supplementary Table 3). CMV serostatus did not affect SARS-CoV-2 shedding rate and peak viral load (data not shown), or the proportion of patients with detectable SARS-CoV-2 at predefined study visits (Supplementary Figure 2).

### Incidence of and Clinical Risk Factors for CMV DNAemia

**Figure 1.**
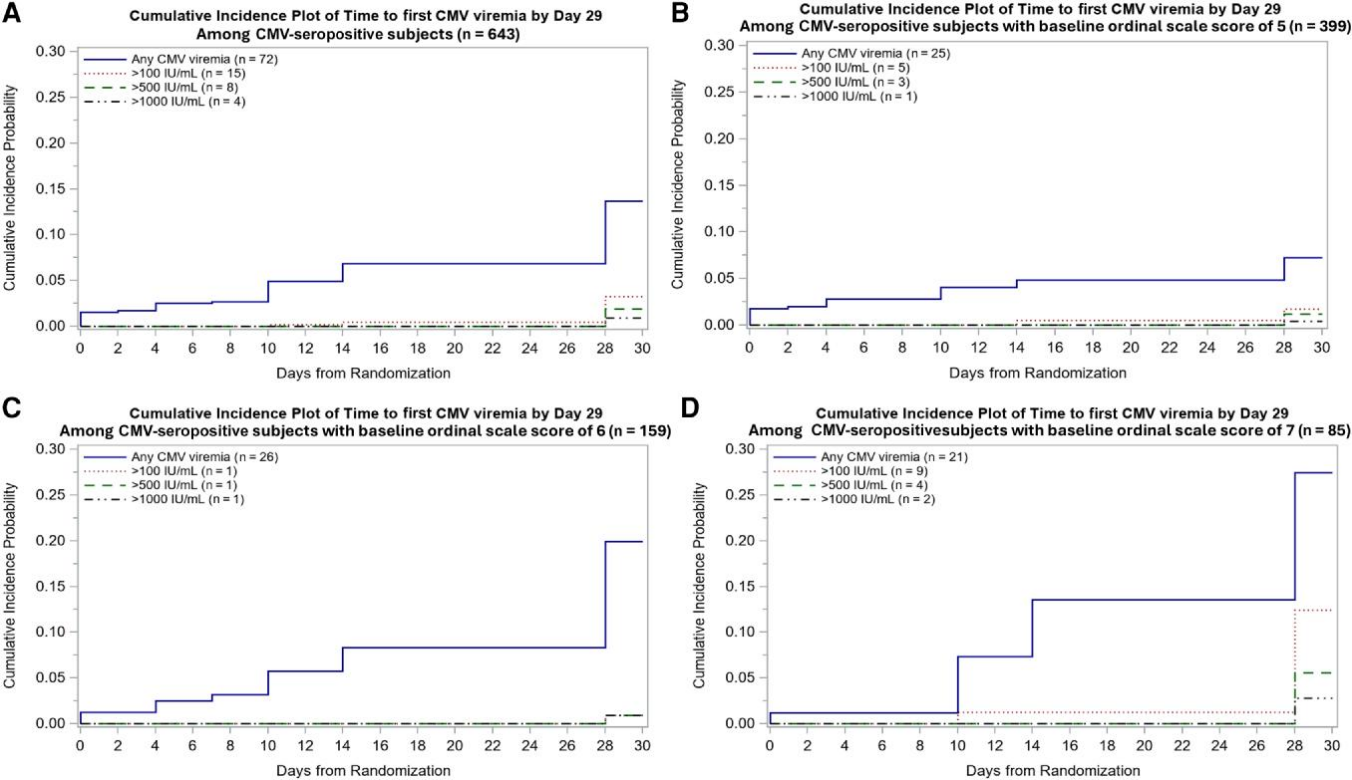
Time to first cytomegalovirus (CMV) reactivation at different viral load thresholds among all CMV-seropositive patients (*A*), and ordinal scale subgroups 5 (*B*), 6 (*C*), and 7 (*D*).

**Figure 2.**
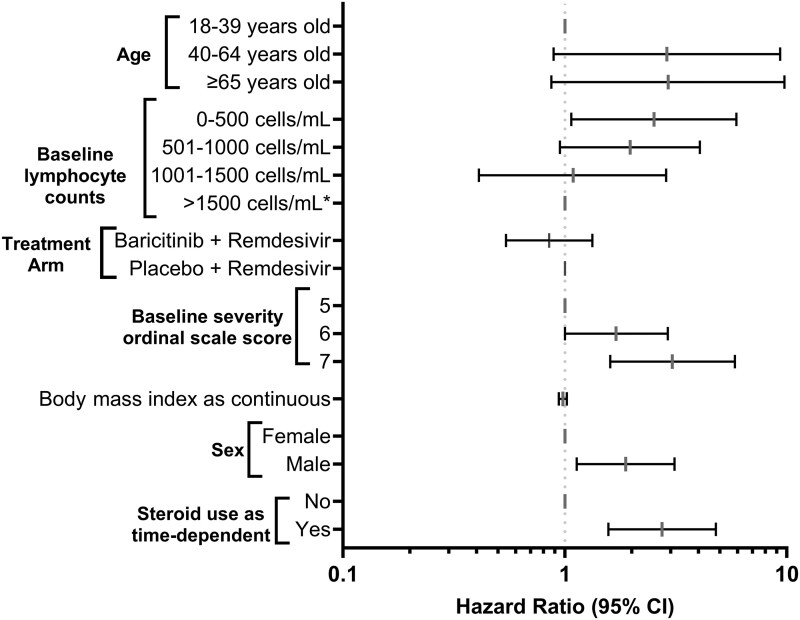
Risk factors for cytomegalovirus reactivation at any level (multivariable analysis). Covariates and category labels are shown on the left axis. Abbreviation: CI, confidence interval.

Plasma CMV DNAemia was measured among 643 CMV-seropositive patients using quantitative PCR at baseline and on days 3, 5, 8, 11, 15, and 29 (N = 2308 samples). The time to first detection at different viremia levels and stratified by OS is shown in Figure 1. Overall, 72 (11.2%) had any detectable DNAemia, 15 (2.3%), 8 (1.2%), and 4 (0.6%) had any CMV >100 IU/mL, >500 IU/mL, and >1000 IU/mL, respectively. Most high-level detections were measured at the day 29 timepoint. The weekly prevalence estimates of CMV DNAemia are shown in Supplementary Figure 2. Risk factors for CMV reactivation included male sex, increasing OS, baseline lymphopenia (absolute lymphocyte count <500 cells/μL), and corticosteroid use (Figure 2). To analyze impact of corticosteroid use on late reactivation, we examined day 29 CMV-seropositive survivors with available CMV and SARS-CoV-2 shedding data (n = 239). While corticosteroid use when analyzed as time-dependent variable was significantly associated with CMV reactivation, two-thirds of patients with late CMV reactivation (at any level [13/19, 68%] and >100 IU/mL [6/9, 67%]) measured at day 28 did not receive corticosteroids after randomization. Randomization to baricitinib + remdesivir versus placebo + remdesivir was not statistically associated with CMV DNAemia (HR, 0.83 [95% CI, .53–1.3]; *P* = .41).

### Impact of DNAemia on Clinical Recovery

**Figure 3.**
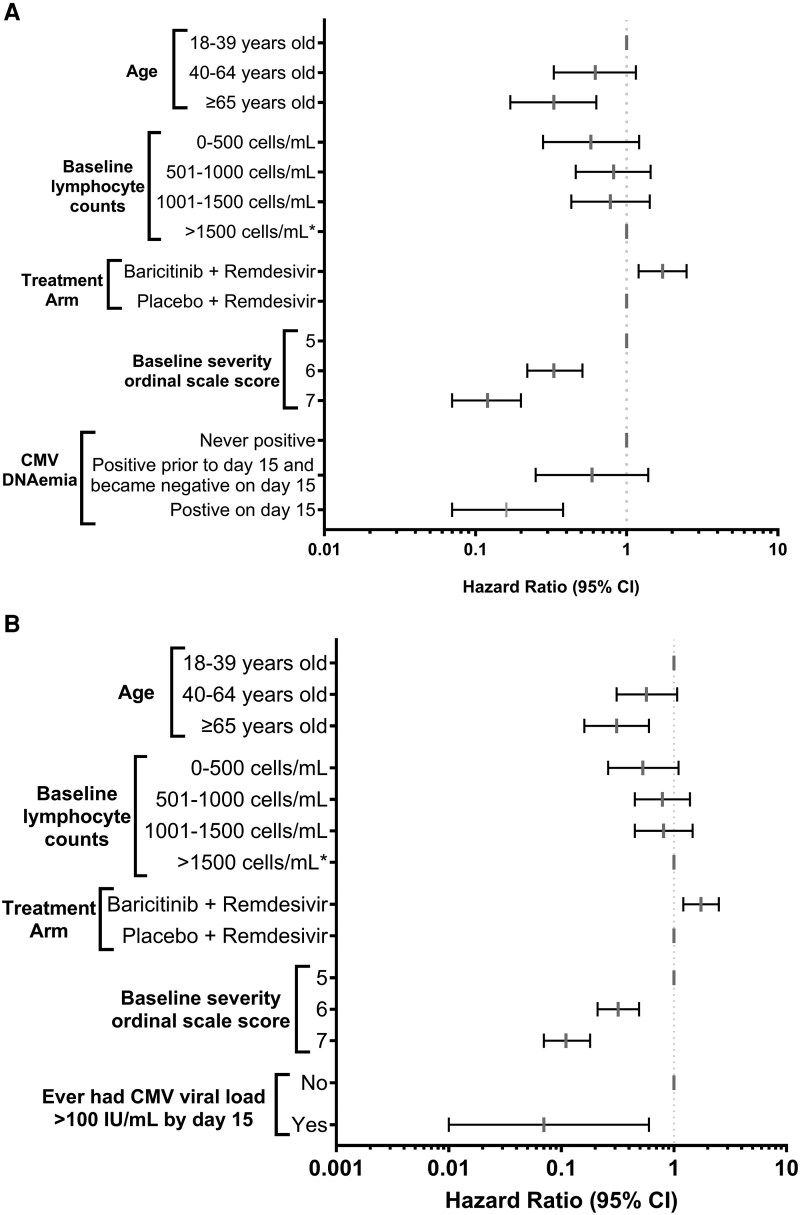
Effect of CMV DNAemia at any level (*A*) and ≥100 IU/mL (*B*) on clinical status at day 29. Results show the probability of having a lower ordinal scale (ie, improved outcome) at day 29. The CMV variable in (*B*) is binary due to low number of events. Abbreviations: CI, confidence interval; CMV, cytomegalovirus.

The association of OS and the impact of baricitinib use on clinical improvement was confirmed in this study population with similar effect size compared to the original clinical trial (Figure 3, Supplementary Figure 3) [17]. CMV DNAemia was associated with a lower probability of clinical improvement at day 29 in adjusted models (Figure 3), with HR for clinical improvement of 0.31 (95% CI, .17–.57; *P* < .001) in those with any CMV DNAemia by day 15 compared to those without measurable CMV DNAemia. The effect was more pronounced with higher CMV viral load (≥100 IU/mL) (HR, 0.07 [95% CI, .01–.60]; *P* = .016), but this analysis was less robust due to the small number of events. To examine the effect of clearance of the CMV viremia on clinical recovery we compared patients who were never viremic with patients who were viremic early but cleared by day 15 and those who were still viremic at day 15 (using the same adjustment variables). Persistent viremia was statistically significant with delayed clinical recovery (Figure 3*A*).

### Impact of CMV DNAemia on Mortality

**Figure 4.**
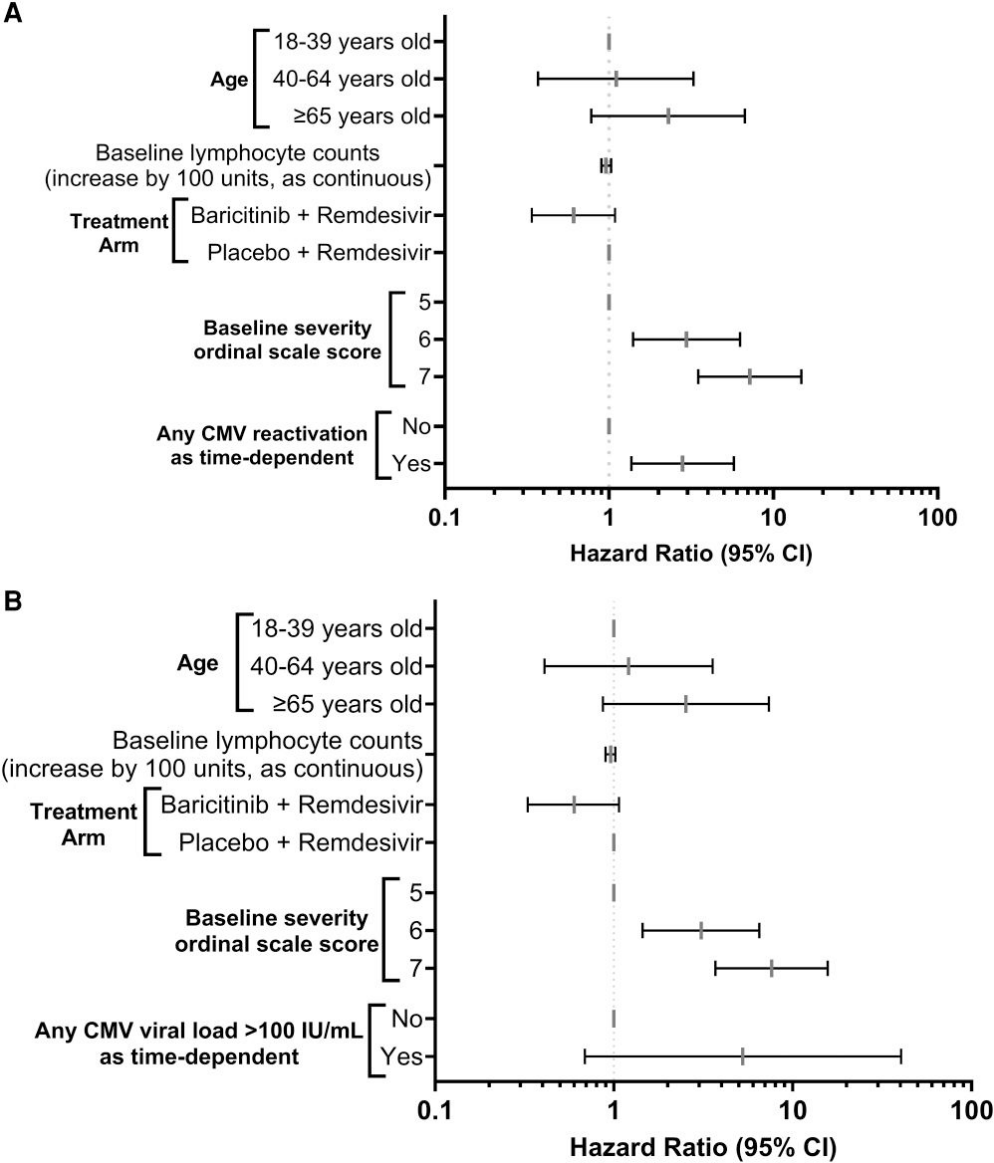
Effect of CMV DNAemia at any level (*A*) and ≥100 IU/mL (*B*) on death by day 29. Abbreviations: CI, confidence interval; CMV, cytomegalovirus.

**Figure 5.**
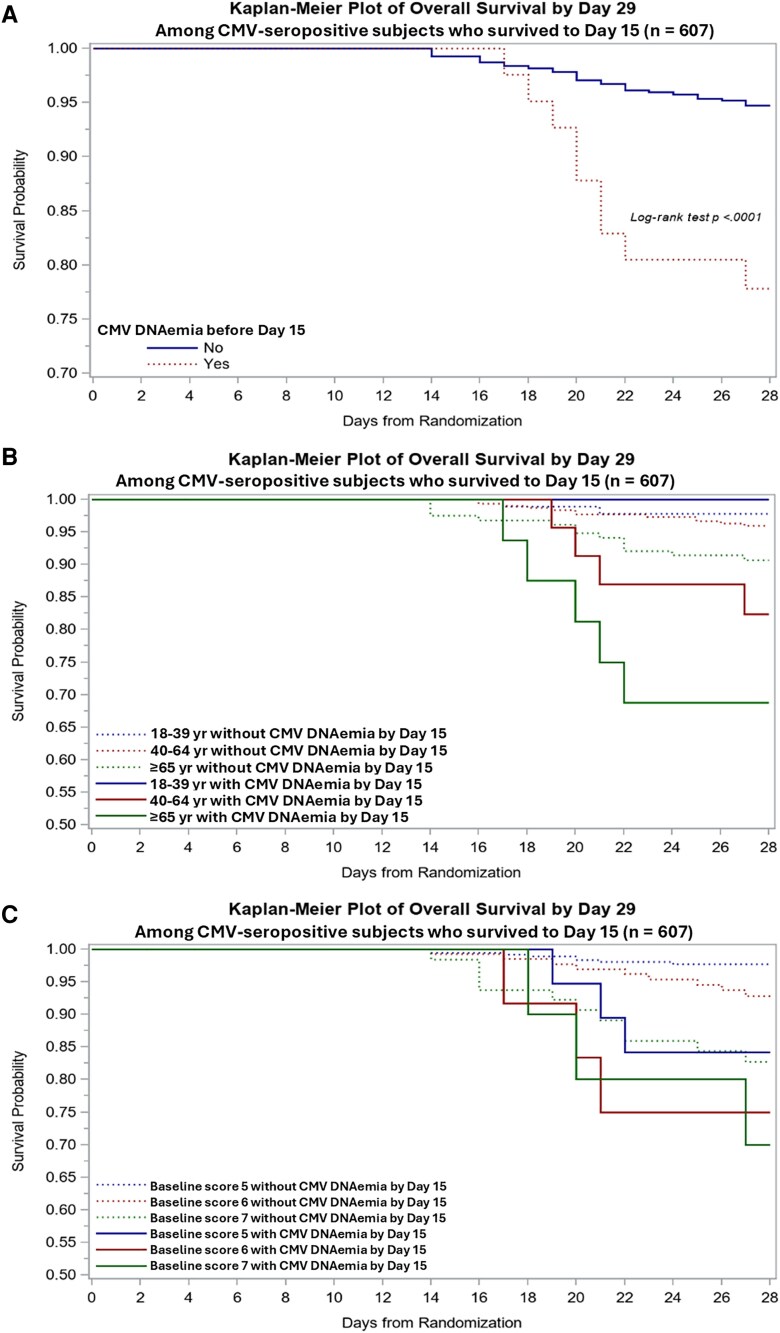
Time to death among cytomegalovirus (CMV)–seropositive day 14 survivors stratified by CMV DNAemia during the first 14 days of study (*A*), DNAemia and age (*B*), and DNAemia and ordinal scale group (*C*).

Any CMV DNAemia was associated with mortality at day 29 in multivariable models. The HR for death was 2.80 (95% CI, 1.37–5.75; *P* = .005) for any CMV DNAemia compared to none, where CMV DNAemia treated as a time-dependent variable. Due to the limited number of events, we performed several models, which consistently showed an association of DNAemia at any level and death (Figure 4*A*). Higher levels of CMV DNAemia were associated with a higher HR of death for CMV DNAemia ≥100 IU/mL compared to none (Figure 4*B*). An additional landmark analysis was performed among day 15 survivors, showing follow-up results consistent with the main analysis. CMV DNAemia at any level during the first 2 weeks of the study was associated with increased mortality in multivariable analyses, HR for death was 3.43 (95% CI, 1.59–7.42; *P* = .002) in for CMV DNAemia at any level compared to none in survivors to day 15 (Supplementary Figure 3). The effect was present across the age spectrum and more pronounced in participants in OS 6 and 7 (Figure 5).

### Impact of CMV DNAemia on SARS-CoV-2 Viral Load Kinetics

**Figure 6.**
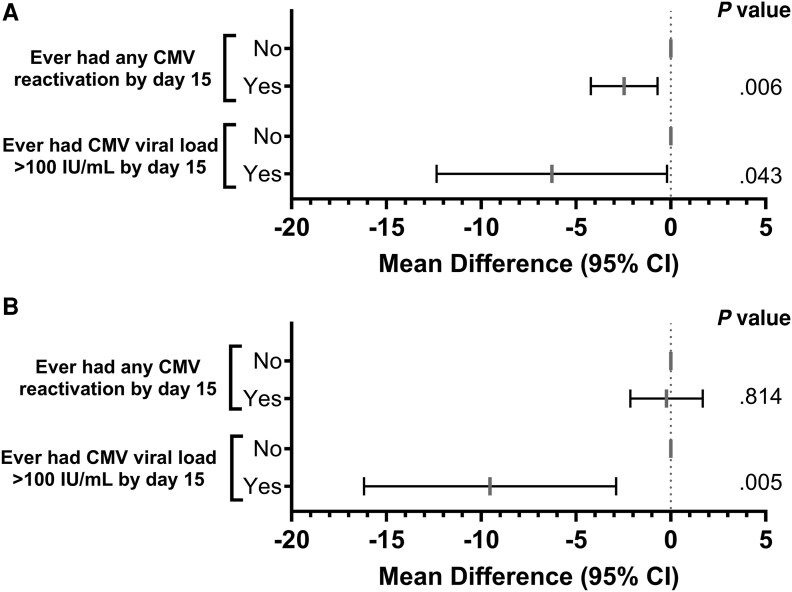
Multivariate analysis of maximum SARS-CoV-2 viral load between baseline and day 15 (*A*) and between day 15 and 29 (*B*) among day 15 survivors. Mean differences refer to cycle threshold values obtained by polymerase chain reaction. Abbreviations: CI, confidence interval; CMV, cytomegalovirus; SARS-CoV-2, severe acute respiratory syndrome coronavirus 2.

In a landmark analysis of day 15 survivors with available SARS-CoV-2 viral load data in nasal specimens (n = 262) who had ongoing SARS-CoV-2 shedding, CMV DNAemia at any level during the first 2 weeks was associated with SARS-CoV-2 higher viral loads at day 15, with an increased effect size with high-level CMV DNAemia (Figure 6*A*). The effect on SARS-CoV-2 shedding was also detectable for high CMV viral loads on day 29 (Figure 6*B*). In a model adjusted for geographic region, CMV DNAemia at any level between baseline and day 15 was associated with a higher risk of detectable SARS-CoV-2 shedding at day 15 (adjusted odds ratio, 2.67 [95% CI, 1.03–6.9]; *P* = .042); no association was detectable with persistent shedding at day 29.

## DISCUSSION

Hospitalization, critical illness, and death associated with COVID-19 continues to be prevalent in 2025, with the severity of illness being similar or exceeding that of influenza virus disease [1–3, 31]. In this study, we showed that CMV DNAemia occurs in 15% of CMV-seropositive SARS-CoV-2–infected hospitalized patients requiring oxygen, with significantly higher incidence in patients with lymphopenia, exposure to corticosteroids, and higher oxygen requirement. CMV DNAemia was independently associated with a delayed clinical improvement, a higher SARS-CoV-2 viral load, and increased mortality—and the higher CMV viral load, the higher the risk of these 3 outcomes. Randomization to baricitinib, an immunomodulatory agent targeting the JAK-STAT pathway, compared to placebo did not result in increased CMV reactivation. Use of immunomodulatory agents for acute COVID-19 risks development of secondary infections or opportunistic infections, but here we demonstrate that this did not occur for CMV.

A possible role of CMV as an effect modifier in the course and outcome of SARS-CoV-2 disease has been hypothesized in the early days of the pandemic [21], and the literature to date indicates that CMV reactivation does in fact occur in hospitalized patients and may be associated with poor outcomes [21–26]. However, the data were largely based on retrospective studies based on convenience samples that analyzed results from clinical testing. Therefore, there was a need for a systematic analysis using prospective datasets with standardized sampling schedules [32]. Here we analyzed both clinical and virologic risk factors for CMV reactivation and its impact on clinical recovery and SARS-CoV-2 viral kinetics. The finding that CMV DNAemia occurs primarily in COVID-19 patients who are critically ill is consistent with what has been observed in non–COVID-19 patients [22–25, 33]. Additional risk factors we identified (lymphopenia, sex, corticosteroid use) are well established in the transplant population [34–36]. For the analysis of clinical outcomes, we used the same primary endpoint of the parent trial—that is, time to clinical recovery. CMV DNAemia delayed time to recovery, with a more delayed recovery seen with higher CMV viral loads, although the analysis of the viral load effect was limited by the relatively small number of patients with high viral load. We also examined whether CMV kinetics were important and found that the association with delayed clinical recovery was primarily seen in patients with persistent viremia at day 15. The study also demonstrated an association of CMV DNAemia with a higher mortality in both univariate and multivariable analyses.

CMV serostatus at baseline did not appear to be important for clinical or virologic outcomes of SARS-CoV-2; however, DNAemia was a strong predictor. A mechanism by which CMV DNAemia delays clinical recovery could be extending SARS-CoV-2 viral replication. Specifically, CMV DNAemia may synergistically enhance the immunosuppressive state, resulting in a further delay of SARS-CoV-2 immune responses and delayed viral clearance [11–13]. Indeed, CMV DNAemia was associated with higher levels of SARS-CoV-2 peak viral load and prolonged shedding (Figure 6). The effect was enhanced with higher CMV viral load. Studies are needed to examine this hypothesis.

The results raise the question whether CMV PCR testing is clinically indicated or whether CMV may potentially be a therapeutic target. CMV reactivation has been observed in critically ill patients without SARS-CoV-2 infections [32, 37], but so far evidence is lacking that treatment or prevention of CMV results in improved management strategies [38]. CMV reactivation could by itself adversely affect outcome through direct or indirect immunomodulatory effects [39–41]. But it could also be merely a bystander, or an inherent part of the inflammatory pathways associated with severe SARS-CoV-2 infection. In a large well-phenotype cohort study, activation of CMV-specific CD8 T cells in the absence of CMV viremia suggest this bystander effect [42]. Another proposed mechanism of CMV-induced immunomodulatory effect could be cross-reactive and dysfunctional CMV-specific T cells [43]. Randomized clinical trials of interventions that suppress or treat CMV are needed to conclusively establish a causative relationship between CMV and clinical outcomes in patients with SARS-CoV-2. The effect of corticosteroids is the only potentially modifiable risk factor; however, whether avoiding steroids or dose reductions would alter the observed associations cannot be determined from this study. Early treatment of SARS-CoV-2 with anti-SARS-CoV-2 drugs or effective monoclonal antibodies in the outpatient setting is a proven strategy to prevent progression to hospitalization and progressive disease [44]. Early treatment of patients at high risk for hospitalization will likely also reduce the risk of developing CMV reactivation, which in turn will mitigate its negative impact as CMV reactivation is strongly associated with higher disease severity based on OS stages.

A key strength of this study is the high-quality design, protocolized sample collection, large sample size, and the validated data of the parent ACTT-2 trial. While the trial tested 2 interventions, neither drug has activity against CMV, and the study arm was not associated with any of the outcomes examined in this study. However, the results associated with baricitinib seen in the original trial could be replicated in the subset of CMV-seropositive patients. The study was conducted in 2020 in unvaccinated study participants when the ancestral strain of SARS-CoV-2 was circulating, which raises the question whether the data are applicable for subsequent variants with current treatment standards. Studies performed in subsequent different variant areas in which vaccination was fully available showed similar benefits to ACTT-2 [45, 46]. In addition, all study participants were treated with an effective antiviral drug, remdesivir, and a retrospective registry study in hospitalized patients from Korea that analyzed data from 2021–2022 when the Delta and Omicron variants were prevalent also showed that CMV occurred in sicker patients and outcome of these patients appeared to be poor, although the sample size was relatively small [38]. Other limitations are that CMV reactivation in other compartments, such as the lung, could not be measured due to unavailability of samples, and that the SARS-CoV-2 viral kinetics analyses could only be conducted in a subset of patients with available data.

In conclusion, CMV reactivation occurred during severe COVID-19 and acted as a disease modifier for multiple relevant COVID-19 outcomes. Corticosteroid use, lymphopenia, male sex, older age, and COVID-19 disease severity were all associated with CMV DNAemia in hospitalized COVID-19 patients requiring supplemental oxygen. CMV reactivation was associated with decreased probability of clinical improvement, prolonged SARS-CoV-2 shedding, higher viral load, and increased mortality in this patient population. Further studies are needed to determine the epidemiology and outcome of CMV with current variants and whether prevention or suppression of CMV can lead to improved management strategies of seriously ill patients infected with SARS-CoV-2. The study also provides the rationale to conduct detailed analyses of the impact of early or recurrent reactivation of CMV on postacute COVID sequelae [47, 48].

## Supplementary Material

jiaf649_Supplementary_Data

## Supplementary Data

Supplementary materials are available at *The Journal of Infectious Diseases* online (http://jid.oxfordjournals.org/). Supplementary materials consist of data provided by the author that are published to benefit the reader. The posted materials are not copyedited. The contents of all supplementary data are the sole responsibility of the authors. Questions or messages regarding errors should be addressed to the author.
