# Supplementary material for: Cytomegalovirus DNAemia in Hospitalized Adults With SARS-CoV-2 Infection Requiring Supplemental Oxygen: Virologic and Clinical Characteristics and Association With Outcomes
Source: J Infect Dis. 2025 Dec 31;233(5):812–22. doi: 10.1093/infdis/jiaf649 (PMC13175614; doi:10.1093/infdis/jiaf649)
Supplement: jiaf649_Supplementary_Data [file jiaf649_supplementary_data.docx]

**Supplementary Material**

Boeckh M, Xie H, Stevens-Ayers T, Sircy L, Zamora D, Goldman JD, Woods CW, Stapleton RD, Rubenfeld G, Kalil A, Jerome KR, Dasgupta S, Limaye AP. Cytomegalovirus DNAemia in Hospitalized Adults with SARS-CoV-2 Infection Requiring Supplemental Oxygen: Virologic and Clinical Characteristics and Association with Outcomes. J Infect Dis.

| **Section** | **Page** |
| --- | --- |
| Supplemental Table S1. National Allergy and Infectious Diseases (NIAID) Ordinal Scale. | 2 |
| Supplemental Table S2. Patient characteristics for patients with available samples for SARS-CoV-2 shedding analysis. | 3 |
| Supplemental Table S3. Multivariable analysis of time to recovery (attaining ordinal scale score 1, 2, or 3) among all patients (N=772). | 4 |
| Supplemental Figure S1. Time to recovery based on ordinal scale among CMV seropositive patients (N=643). | 5 |
| Supplemental Figure S2. Proportional detection of SARS-CoV-2 in oropharyngeal, nasopharyngeal or nasal swabs by study visit day and CMV serostatus in those (n=543, participants with available shedding data). R+ and R- indicate CMV seropositive and seronegative, respectively. | 6 |
| Supplemental Figure S3. Effect of CMV DNAemia at any level (A) and >100 IU/mL (B) on death by day 29 (landmark analysis of day 15 survivors). | 7 |

**Supplemental Table S1. National Allergy and Infectious Diseases (NIAID) Ordinal Scale.**

| Recovered | 1 | Not hospitalized, no limitations on activities |
| --- | --- | --- |
|  | 2 | Not hospitalized, limitation on activities and/or requiring home oxygen |
|  | 3 | Hospitalized, not requiring supplemental oxygen – no longer requires ongoing medical care |
|  | 4 | Hospitalized, not requiring supplemental oxygen – requiring ongoing medical care |
| Population Included | 5 | Hospitalized, requiring supplemental oxygen |
|  | 6 | Hospitalized, on non-invasive ventilation or high flow oxygen devices |
|  | 7 | Hospitalized, on mechanical ventilation or ECMO |
|  | 8 | Death |

**Supplemental Table S2.** Patient characteristics for patients with available samples for SARS-CoV-2 shedding analysis.

| **Variables** | **Categories** | **Overall Cohort  (N=891)** | **CMV+ Subjects  (N=487)** | **CMV+/- Subjects  (N=593)** | **CMV+/- Subjects  (N=325)** |
| --- | --- | --- | --- | --- | --- |
| Age (years) | Mean (std) | 55.19 (14.63) | 55.43 (14.13) | 54.97 (14.52) | 55.74 (14.81) |
|  | Median (range) | 56.00 (18.00-89.00) | 57.00 (18.00-88.00) | 56.00 (18.00-88.00) | 57.00 (18.00-88.00) |
|  |  |  |  |  |  |
| Randomized Treatment | Baricitinib + Remdesivir | 445 (50%) | 253 (52%) | 301 (51%) | 167 (51%) |
|  | Placebo + Remdesivir | 446 (50%) | 234 (48%) | 292 (49%) | 158 (49%) |
|  |  |  |  |  |  |
| BMI (kg/m2) | Mean (std) | 32.45 (8.26) | 32.27 (7.82) | 32.60 (8.04) | 32.46 (7.95) |
|  | Median (range) | 31.00 (16.10-91.90) | 31.10 (16.10-81.20) | 31.25 (16.10-81.20) | 31.30 (16.10-66.80) |
|  |  |  |  |  |  |
| Sex | F | 325 (36%) | 184 (38%) | 215 (36%) | 124 (38%) |
|  | M | 566 (64%) | 303 (62%) | 378 (64%) | 201 (62%) |
|  |  |  |  |  |  |
| Race | AMERICAN INDIAN | 7 (1%) | 4 (1%) | 7 (1%) | 4 (1%) |
|  | ASIAN | 64 (7%) | 33 (7%) | 36 (6%) | 20 (6%) |
|  | BLACK OR AFRICAN AMERICAN | 133 (15%) | 73 (15%) | 87 (15%) | 39 (12%) |
|  | NATIVE HAWAIIAN/PACIFIC ISLANDER | 10 (1%) | 4 (1%) | 4 (1%) | 4 (1%) |
|  | UNKNOWN | 239 (27%) | 128 (26%) | 142 (24%) | 75 (23%) |
|  | WHITE | 438 (49%) | 245 (50%) | 317 (53%) | 183 (56%) |
|  |  |  |  |  |  |
| Geographic Region 1 | Non-US Site | 113 (13%) | 18 (4%) | 19 (3%) | 11 (3%) |
|  | US Site | 778 (87%) | 469 (96%) | 574 (97%) | 314 (97%) |
|  |  |  |  |  |  |
| Randomized Disease Severity Stratum | Moderate Disease | 524 (59%) | 287 (59%) | 359 (61%) | 180 (55%) |
|  | Severe Disease | 367 (41%) | 200 (41%) | 234 (39%) | 145 (45%) |
|  |  |  |  |  |  |
| Baseline Severity Ordinal Scale Score | 5 | 564 (63%) | 292 (60%) | 365 (62%) | 183 (56%) |
|  | 6 | 216 (24%) | 124 (25%) | 149 (25%) | 80 (25%) |
|  | 7 | 111 (12%) | 71 (15%) | 79 (13%) | 62 (19%) |
|  |  |  |  |  |  |
| Duration of Symptoms at Randomization | Mean (std) | 8.63 (4.54) | 8.45 (4.44) | 8.53 (4.47) | 7.99 (3.99) |
|  | Median (range) | 8.00 (0.00-35.00) | 8.00 (0.00-35.00) | 8.00 (0.00-35.00) | 8.00 (0.00-32.00) |
|  |  |  |  |  |  |
| Steroid use at Baseline | 0 | 815 (91%) | 435 (89%) | 530 (89%) | 289 (89%) |
|  | 1 | 76 (9%) | 52 (11%) | 63 (11%) | 36 (11%) |
|  |  |  |  |  |  |
| Dexamethasone Products use at Baseline | 0 | 854 (96%) | 464 (95%) | 566 (95%) | 310 (95%) |
|  | 1 | 37 (4%) | 23 (5%) | 27 (5%) | 15 (5%) |
|  |  |  |  |  |  |
| ALC at Baseline | 1 | 83 (10%) | 40 (8%) | 48 (8%) | 30 (9%) |
|  | 2 | 363 (42%) | 207 (43%) | 251 (42%) | 152 (47%) |
|  | 3 | 269 (31%) | 140 (29%) | 178 (30%) | 92 (28%) |
|  | 4 | 154 (18%) | 96 (20%) | 111 (19%) | 47 (14%) |
|  | 5 | 4 (<1%) | 3 (1%) | 4 (1%) | 4 (1%) |

**Supplemental Table S3.** Multivariable analysis of time to recovery (attaining ordinal scale score 1, 2, or 3) among all patients (N=772).

| **Covariates** | **Categories** | **Hazard Ratio (95% CI)** | **P-values** |
| --- | --- | --- | --- |
| Age | 18 - <40 yr | 1 |  |
|  | 40 - <65 yr | 0.75 (0.61-0.92) | 0.007 |
|  | >=65 yr | 0.53 (0.40-0.68) | <.001 |
| Baseline lymphocyte counts | 0 - 500 | 0.64 (0.46-0.90) | 0.009 |
|  | >500 - 1000 | 0.70 (0.56-0.87) | 0.001 |
|  | >1000 - 1500 | 0.79 (0.63-1.00) | 0.048 |
|  | >1500* | 1 |  |
| Treatment Arm | Baricitinib + Remdesivir | 1.28 (1.09-1.49) | 0.002 |
|  | Placebo + Remdesivir | 1 |  |
| Baseline Severity Ordinal Scale Score | 5 | 1 |  |
|  | 6 | 0.44 (0.36-0.53) | <.001 |
|  | 7 | 0.16 (0.12-0.22) | <.001 |
| Body-mass index | as continuous | 1.01 (1.00-1.02) | 0.215 |
|  |  |  |  |
|  |  |  |  |
| Ethnic group | Hispanic or Latino | 1 |  |
|  | Not Hispanic or Latino | 0.93 (0.78-1.10) | 0.38 |
|  | Not Reported or Unknown | 0.44 (0.25-0.77) | 0.004 |
| Baseline CMV serostatus | Negative | 1 |  |
|  | Positive | 0.91 (0.73-1.12) | 0.368 |

* Including missing values.

**Supplemental Figure S1.** Time to recovery based on ordinal scale among CMV seropositive patients (N=643).


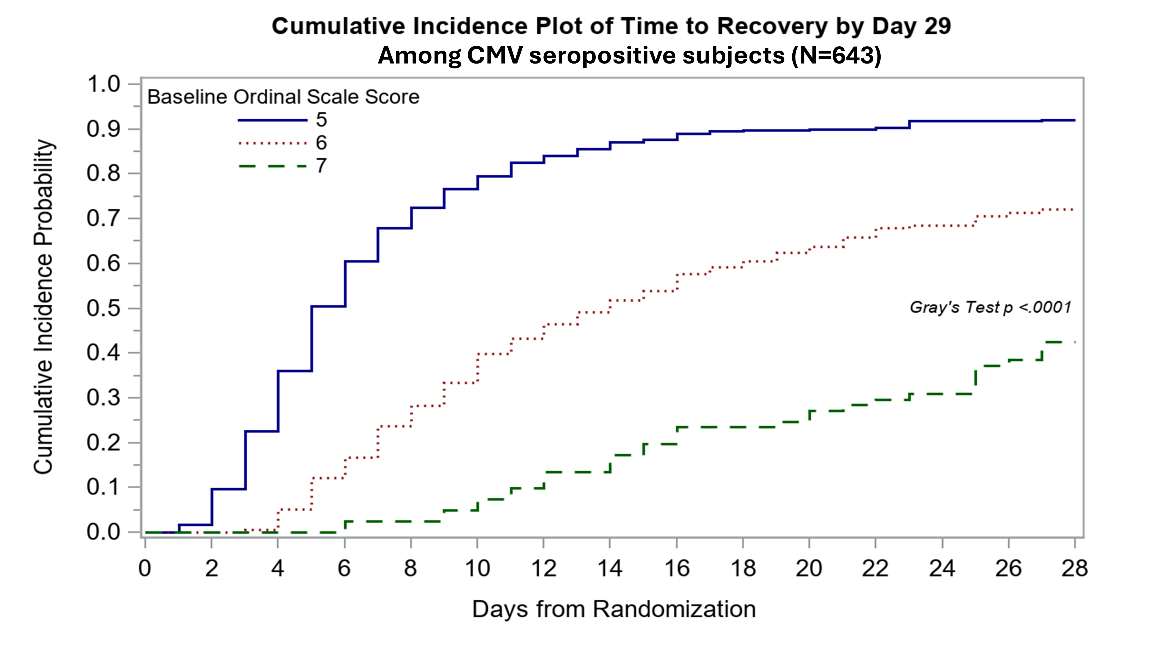


**Supplemental Figure S2.** Proportional detection of SARS-CoV-2 in oropharyngeal, nasopharyngeal or nasal swabs by study visit day and CMV serostatus in those (n=543, participants with available shedding data)**.**  R+ and R- indicate CMV seropositive and seronegative, respectively.


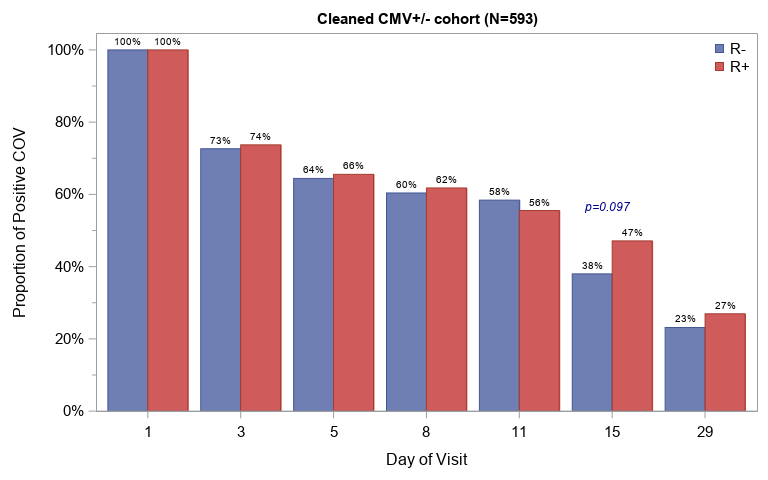

**Supplemental Figure S3**. Effect of CMV DNAemia at any level (A) and >100 IU/mL (B) on death by day 29 (landmark analysis of day 15 survivors).
